# Suppression of radiation-induced point defects by rhenium and osmium interstitials in tungsten

**DOI:** 10.1038/srep36738

**Published:** 2016-11-08

**Authors:** Tomoaki Suzudo, Akira Hasegawa

**Affiliations:** 1 Center for Computational Science and e-Systems, Japan Atomic Energy Agency 2-4 Shirane Shirakata Tokai-mura, 319-1195, Japan; 2 Department of Quantum Science and Energy Engineering, Tohoku University 6-6-01-2 Aramaki-aza-Aoba Aoba-ku Sendai, 980-8579, Japan

## Abstract

Modeling the evolution of radiation-induced defects is important for finding radiation-resistant materials, which would be greatly appreciated in nuclear applications. We apply the density functional theory combined with comprehensive analyses of massive experimental database to indicate a mechanism to mitigate the effect of radiation on W crystals by adding particular solute elements that change the migration property of interstitials. The resultant mechanism is applicable to any body-centered-cubic (BCC) metals whose self-interstitial atoms become a stable crowdion and is expected to provide a general guideline for computational design of radiation-resistant alloys in the field of nuclear applications.

For the breakthrough of next-generation nuclear reactors development, understanding physics of radiation-induced defects in metals and alloys is critical^1^,^2^, and a central issue of the research is how to design radiation-resistant materials^3^. Tungsten (W) is a promising candidate of plasma-facing materials (PFM) in fusion reactors because it has high melting temperature, high resistance to sputtering, and high thermal conductivity^4^. However, radiation-induced vacancies and their clusters generated in W materials seem to trap hydrogen isotopes such as deuterium^5^, and the feasibility of PFM made of W materials is still questionable because such retention may seriously deteriorate safety and efficiency of the fusion devices.

Some solute elements such as rhenium (Re) and osmium (Os) become aggregated in W crystals and become precipitated causing loss of ductility as the second phase under neutron irradiation^6^; this is called radiation-induced precipitation (RIP). Even in pure W, Re and Os are produced under irradiation through nuclear transmutation and become precipitated. One can think of a few possible mechanisms for RIP. For example, extended defects such as dislocations or grain boundaries might provide nucleation sites for aggregation, but an experiment^7^ shows that the aggregation occurs without extended defects. Another hypothesis is that vacancy or interstitial clustering might cause the aggregation, but the RIP is not correlated to the growth of voids and dislocation loops^8^. The remaining possibility seems to be that it is caused by the recombination of a vacancy and an interstitial, both of which are associated with the solute elements^9^,^10^. Table 1 shows the binding energies of solute atoms to point defects, and it clearly indicates that all the combinations of solute elements and point defects are attractive, including Re in Molybdenum (Mo) crystals that also causes RIP^11^. Note that other solute atoms are not necessarily attractive to the point defects in W crystals^12^. It is also known that inclusion of Re and Os into W crystals suppresses swelling^13^ and void growth^14^; such experiments suggest that Re or Os addition makes the material radiation-resistant. In addition, a recent study^15^ made another discovery that inclusion of Re reduces deuterium retention associated with the suppression of surviving vacancies; this is demonstrated by the fact that positron lifetime for W-Re alloys hardly increase after Fe-ion irradiation^15^. This implies that Re and Os not only cause RIP but also enhance vacancy-interstitial recombination that probably causes the suppression of void swelling.

In this paper we would like to uncover mechanism of the enhanced recombination within cascade displacement influenced region, which would be greatly helpful for improving the performance of the fusion devices. Our previous studies^10^,^16^ investigate stability and mobility of Re and Os interstitials; these solutes form a mixed dumbbell with a W atom and have three-dimensional (3D) motion instead of one-dimensional (1D) one that self-interstitial atoms (SIAs) have. We formulated a hypothesis that the change of migration dimension might be related to the suppression of radiation effects. In the following, we revisit the study of the migration path of these solute interstitials because the stability of SIA and Re interstitial is still under debate since a previous first principles study^16^ implies that some uncommon configuration, called a 〈11 h〉 dumbbell^16^ or a bridge dumbbell^17^, may be the most energetically favored. By taking the uncommon dumbbell into consideration, we revised the picture of interstitial migration in W crystals. The trajectories of the interstitials were examined over a wide range of temperatures for the discussion of the enhanced recombination by using a unique statistical method as explained in the following. In addition, we extended the study to Mo crystals that have body-centered-cubic (BCC) structure such as W crystals have and that cause RIP when alloyed with Re; this extended study was useful for supporting our hypothesis and excluding other likely mechanisms of the enhanced recombination. To compensate the above theoretical analyses and to reach the final conclusion, massive experimental data including ones released after our previous studies were thoroughly examined because even state-of-arts modeling and computing technologies are still short of accurate prediction of radiation effects.

## Results

### First principles analyses

**Figure 1:**
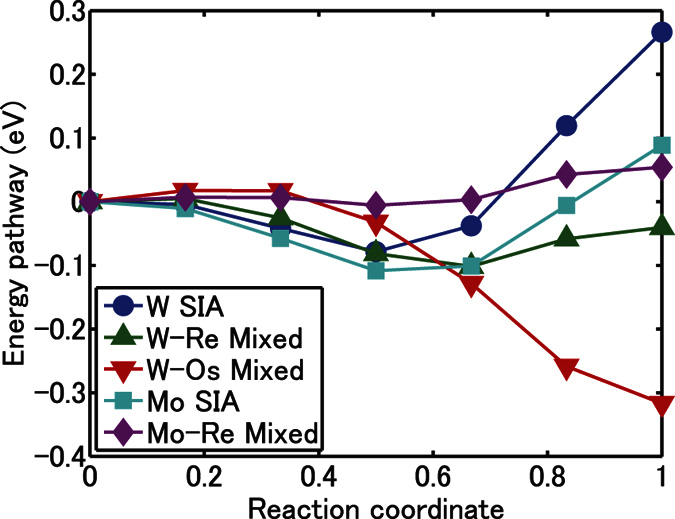
Energy pathways from 〈111〉 to 〈110〉 dumbbells for SIA dumbbells and mixed dumbbells in W and Mo: The values are evaluated by the first principles DFT calculations combined with nudged elastic band method.

**Figure 2:**
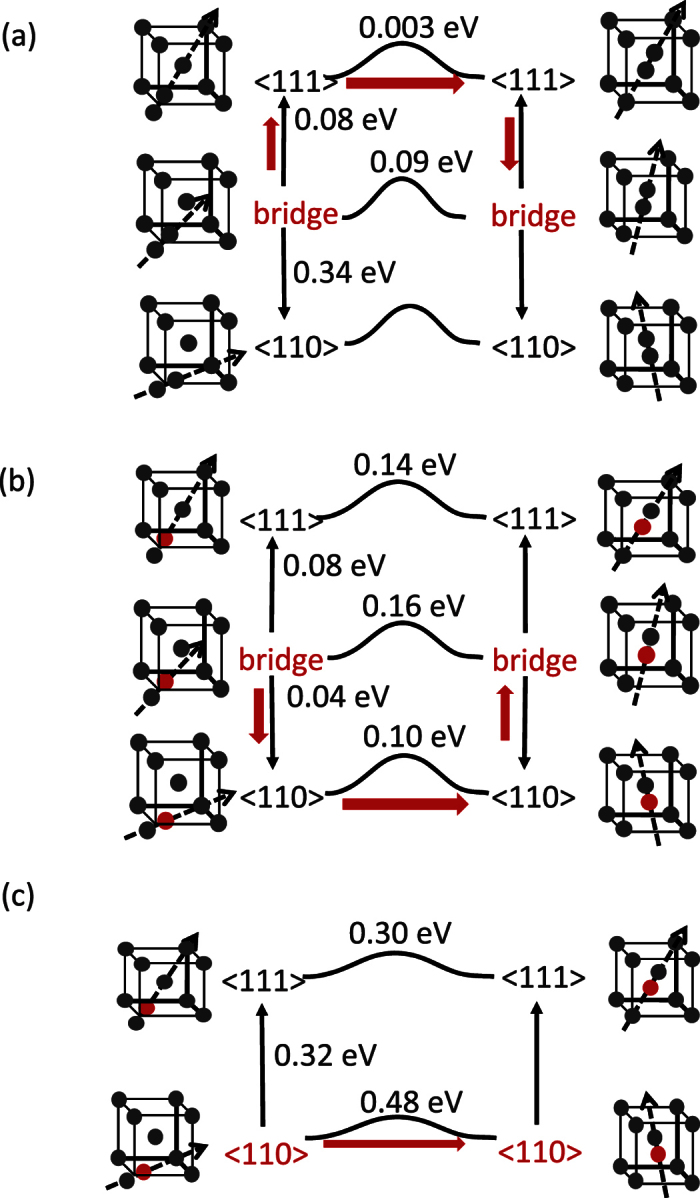
Energetic landscape given by DFT calculations for (**a**) SIA, (**b**) W-Re mixed dumbbell and (**c**) W-Os mixed dumbbell: Black and red spheres indicate solvent and solute atoms, respectively; each thin arrow points the configuration with higher formation energy; each thick and red arrow indicates the migration path of the interstitials from the most favored configuration written by red letters to that at the first nearest neighborhood through the lowest migration barrier.

For the numerical methods the density functional theory (DFT) is applied to conduct the first principles calculations. Through this method energy pathways between 〈111〉 and 〈110〉 configurations of the pure and mixed dumbbells in W crystals are given in Fig. 1. The barriers for these pure/mixed dumbbells, for jumping to a first nearest neighboring (1NN) site, are also given as shown in Fig. 2. As seen in Fig. 1, the most energetically favored SIA in W crystals is a bridge dumbbell that has an intermediate direction between 〈111〉 and 〈110〉. As shown in Fig. 2a when the bridge dumbbell migrates, it rotates to a 〈111〉 direction and migrate to a 1NN site and regain a bridge-dumbbell direction. This is different from a common crowdion motion; this “crowdion” is not straight and it fluctuates when migrating. This migration mode may be recognized only in DFT calculations because experiments cannot observe such a subtle motion. The migration barrier of SIAs is ~0.08 eV (see thick arrows in Fig. 2a) because the rotation barrier from bridge to 〈111〉 direction must be added to a simple crowdion motion barrier, and it is much larger than one evaluated by previous first principles calculations, e.g., 0.0026 eV^18^. Recent experiments, however, shows that the migration barrier can be as large as 0.085 eV^19^ or 0.020 eV^20^. Such discrepancies from the previous DFT results seem to be explained by our novel finding of the fluctuating crowdion motion. It is worth noting that the same final conclusion is given by migration barrier between 0.0026 eV and 0.08 eV, and we adopt the migration barrier of 0.08 eV in the following numerical analysis. For SIAs to change their migration direction they need to rotate from one bridge dumbbell to another by surpassing the neighboring 〈110〉 direction. As shown in Fig. 2a, this energy barrier is 0.34 eV, and the rotation event may occur frequently at high temperatures, suggesting that SIA motion may not be 1D but 3D. This matter will be examined in the following by using a kinetic model.

The most energetically favored W-Re mixed dumbbell is also a bridge dumbbell, although the depression of the energy pathway in Fig. 1 is pretty shallow. A bridge W-Re mixed dumbbell is most likely to rotate to the 〈110〉 dumbbell in the course of jumping to a 1NN site (see Fig. 2b). For the rotation from one bridge dumbbell to another, it must pass a 〈111〉 direction and this energy barrier is 0.08 eV. An Os interstitial is stabilized as a 〈110〉 mixed dumbbell as seen in Fig. 1 and jumps to a 1NN site where it forms another 〈110〉 dumbbell. For the rotation from one 〈110〉 dumbbell to another it must pass a 〈111〉 direction, the rotation energy barrier for that being 0.32 eV. As summarized in Fig. 2 both W-Re and W-Os mixed dumbbells have the rotation barrier being less than the migration barrier; this suggests they practically have 3D motion.

### Atomic kinetic Monte Carlo analyses

**Figure 3:**
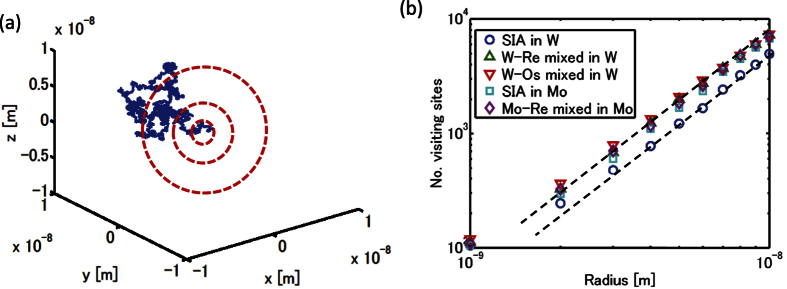
(**a**) The trajectories of the pure/mixed dumbbells are numerically simulated by atomic kinetic Monte Carlo (AKMC) method, and total number of points visited by the trajectory, *N*_*visit*_, is counted inside each sphere in the simulation box (**b**) *N*_*visit*_ as a function of the radius of spheres: Dashed lines show the power of two, which corresponds to the fractal dimension of general 3D random walks.

**Figure 4:**
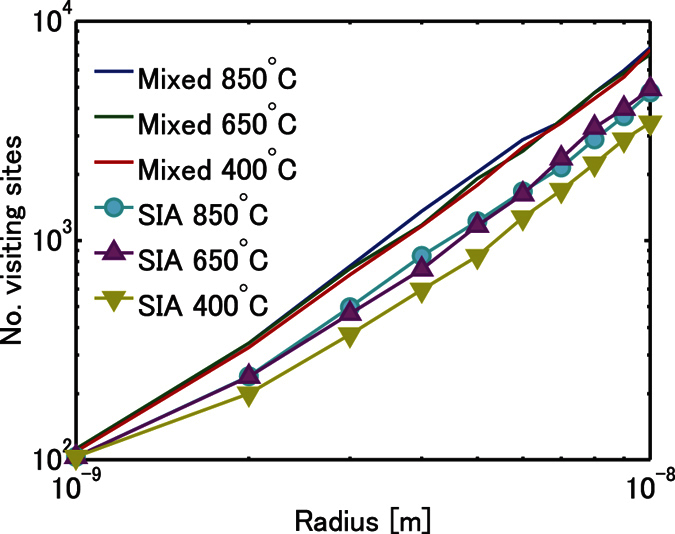
Temperature dependence of *N*_*visit*_ as a function of the radius of spheres for W-Re mixed dumbbells and SIAs in W crystals.

For more detailed analyses, the trajectories of these pure/mixed dumbbells are numerically simulated by atomic kinetic Monte Carlo (AKMC) method. The temperature of the AKMC is 750 °C and is chosen from the experiments^14^ we mainly compare with. We calculate the ‘density’ of the trajectory, i.e., the number of lattice points with any of their eight 1NN sites visited by the migrating objects. This number, *N*_*visit*_, is proportional to the probability of recombination to an isolated vacancy, if the vacancy is immobile. Although vacancies in W crystals are mobile, this assumption is realistic because the migration barrier is 1.69 eV^16^, much larger than the interstitials. *N*_*visit*_ is sliced by the radius of a sphere in the simulation box and is expressed in a double logarithmic plot as seen in Fig. 3. Note that this is data processing for evaluating fractal dimension. If a trajectory is a 3D random walk, its fractal dimension (i.e. the power of this relation) must become two^21^. As one can see in Fig. 3b, the power of each graph asymptotically converges to two as the radius becomes larger; this means that all the trajectories have 3D motion when the resolution of this geometrical analysis is large. Notice that *N*_*visit*_ values for SIAs in W crystals are, meaningfully, smaller than the others, indicating that this trajectory is sparser than the others. The difference is caused by a specific feature of SIAs in W crystals, that is, they have microscopically 1D motion and intermittently change their direction by rotation events^10^. Consequently, they have less chance of recombining with a vacancy compared with W-Re and W-Os mixed dumbbells and have greater chance of escaping the cascade-displacement influenced region. This is an example of the production bias effect^22^, which plays a main role in radiation effect through 1D motion of point defects and their clusters. When Re and Os atoms are present, most interstitials produced by radiation displacement form 3D-migrating mixed-dumbbells, and the production bias effect disappears. The results here imply that the addition of Re or Os enhances vacancy-interstitial recombination probability and decreases the total number of surviving radiation-induced point defects. We also checked the temperature dependence of the above results. As shown in Fig. 4, *N*_*visit*_ for SIAs are meaningfully smaller over several hundreds of degrees.

Mo belongs to the same group in the periodic table as W does, and its chemical properties are similar. Neutron radiation experiments^11^ indicate that addition of Re to Mo crystals also causes RIP, but it does not lead to major suppression of void formation. As seen in Fig. 3b, *N*_*visit*_ values for Mo SIAs and Mo-Re mixed dumbbell are almost identical. The reduction of the production bias by adding Re is not effective for Mo crystals, because SIAs in Mo also have low rotation barrier (Fig. 1) and are also 3D migrating species. The results here also support our hypothesis that reduction of the production bias effect reduces radiation-induced defects. So far the numerical analyses indicates that the increase of *N*_*visit*_ by adding Re or Os to W crystal is a possible cause of the reduction of radiation-induced vacancies. In the rest of this paper, we will check all the other hypothetical causes from experimental database.

## Discussion

Another possible cause of the defect reduction could be the precipitates in W-Re alloys^13^; i.e. vacancies are consumed when forming the second phase of precipitates (i.e. *χ*-phase and *σ*-phase), or interfaces of these second phase could become convenient sites for vacancy-interstitial recombination. These influences, if any, are not significant because suppression of void formation is not distinct in Mo-Re alloys also causing RIP^11^. Besides, the suppression of void formation in W-Re alloys takes place even without RIP^23^. Thus it is possible to narrow down the possibilities, i.e. the main cause of the vacancy suppression is located not at the second phases but at the BCC phase, which can be categorized into two regions, vicinity of extended defects (i.e. grain boundaries and dislocations) and the rest. Atom probe tomography analysis of W-Re alloy after ion irradiation by Xu *et al*.^7^ shows the majority of aggregated Re atoms are in the bulk region. The carriers of Re atoms are vacancies, interstitials and their clusters. The RIP in W-Re alloys usually takes place in association with void suppression, and it forms at high temperatures without interstitial-loop (I-loop) formation as shown by an experiment^8^. These experimental evidences indicate that vacancy-interstitial recombination takes place where the solute atoms aggregate, otherwise the aggregation would have caused growth of vacancy or interstitial clusters, which we do not necessarily see from experiments. So the main location of the vacancy suppression must be in the BCC bulk region, which has only four kinds of defects; single vacancies, vacancy clusters, single interstitials and interstitial clusters. Because a vacancy reaction with another vacancy or their clusters does not cause void suppression that is always associated with Re or Os addition, the remaining possible reactions to reduce vacancies are between a single vacancy and a single interstitial and between a single vacancy and an interstitial cluster. In fact, another kind of production bias effect is caused by 1D motion of interstitial clusters or I-loops, i.e., if migration barriers of I-loops increase due to solute atoms, the I-loops become slower and stay longer in the bulk; this promotes vacancy annihilation and leads to suppression of point defects. This is recognized as the mechanism of radiation swelling suppression observed in Fe-Cr alloys^24^, and is associated with the increase in I-loop density^25^. However, the suppression of void growth by adding Re to W crystals is still observed at high temperatures where no I-loop is formed^8^, so the mechanism discovered in Fe-Cr alloys seems irrelevant in W-Re alloys. Consequently, annihilation of vacancies at isolated interstitials seems to be a remaining possible explanation for the vacancy suppression observed in the experiment^15^. On top of that, all the above numerical analyses support this mechanism.

So far, we checked all the conceivable scenarios of the problem by the combination of numerical methods and massive experimental data analyses and came to the conclusion that the change of migration property of interstitials seems to be the only effective cause of the vacancy suppression. The resultant mechanism is applicable to any BCC metals whose SIA becomes a stable crowdion, such as to V, Nb and Ta^26^, and is applied to designing radiation-resistant alloys.

## Methods

### First Principles methods

The density functional theory (DFT) is applied to conduct the first principles calculations in the framework of generalized gradient approximation with projector-augmented wave pseudo-potentials^27^ using the Vienna *ab initio* simulation package (VASP)^28^. We, within this framework, adopt Perdew-Burke-Ernzerhof pseudo-potentials^29^ from the VASP library. For all DFT calculations, we use a cut-off energy of 350 eV for the plane-wave basis. Supercells applied are composed of 250 body-centered-cubic lattice sites, having a dimension of (5a × 5a × 5a), respectively, where *a* is the lattice constant. For the supercells, the Monkhorst-Pack 3 × 3 × 3 k-point meshes^30^ is used to sample the Brillouin zone. The periodic boundary condition is applied to all three directions for each case. In each DFT calculation a total energy is obtained: Equations to calculate binding energy values from total energy values are given in Supplementary Information. Each migration energy is defined as the energy barrier between two stable atomic configurations. To evaluate this, the energy pathway between these two states is calculated using the nudged elastic band method, whose functionality is fully embedded in VASP. For all energy barrier calculations, we considered five intermediate points (or images) along the pathway. Gharaee and Erhart^17^ give a systematic investigation how the formation energy of solute interstitial in W crystals changes in different size of super cells. Their results indicate that the estimated values increase as the system size increases, and that the increase rate hardly varies with types of interstitials. As mentioned above the migration energy is evaluated by the difference between two formation energy values for different configuration of interstitials. So large part of the system size dependence is canceled out by this subtraction, and the system size effect of the migration energy would not be significant.

### Kinetic Monte Carlo methods

We analyze the kinetics of the pure/mixed dumbbells by using event-driven atomic kinetic Monte Carlo (AKMC) method^10^. Our purpose is to analyze the trajectory of these dumbbells and to verify that the mixed dumbbells enhance vacancy-interstitial recombination compared with the pure dumbbells. We treat one mobile interstitials in the simulation box and let the defect migrate, based on the migration and rotation paths given above. As shown in Fig. 2, there co-exist two paths of jumping to a 1NN site with similar energy threshold in case of SIAs and W-Re mixed dumbbells. Our AKMC always select the easiest path indicated by the thick arrows in Fig. 2, but the results given above are little influenced by the choice of these paths. We ignore any events of these mixed dumbbells dissociating into an SIA and a solute substitutional because solute substitutionals are not able to migrate, as there is no vacancy in the simulation box. To conduct the AKMC and its post analyses shown in Fig. 3, we use a programming code specially developed for this purpose using a KMC development tool called PAKSS^10^; this is open source software developed by the first author. We set the attempt frequency of 10^13^ *s*^−1^ for any events. We run this AKMC simulation until the defect deviates from the initial position by more than 10^−8^ m, which is similar to the size of cascade-displacement-influenced region^31^. For all the type of mobile interstitials we conduct 128 AKMC runs using different series of random numbers, and mean values of *N*_*visit*_ are used for the analyses in Fig. 3.

## Additional Information

**How to cite this article**: Suzudo, T. and Hasegawa, A. Suppression of radiation-induced point defects by rhenium and osmium interstitials in tungsten. *Sci. Rep.* **6**, 36738; doi: 10.1038/srep36738 (2016).

**Publisher’s note:** Springer Nature remains neutral with regard to jurisdictional claims in published maps and institutional affiliations.

## Supplementary Material

Supplementary Information

## Figures and Tables

**Table 1:** Binding energy of solute atoms to vacancy and SIA in W and Mo crystals (eV): The values are evaluated by the first principles DFT calculations.

Solute atom	Vacancy	SIA
Re-sub in W	0.22	0.74
Os-sub in W	0.52	1.93
Re-sub in Mo	0.10	0.07
