# Supplementary material for: Suppression of radiation-induced point defects by rhenium and osmium interstitials in tungsten
Source: Sci Rep. 2016 Nov 8;6:36738. doi: 10.1038/srep36738 (PMC5099857; doi:10.1038/srep36738)
Supplement: Supplementary Information [file srep36738-s1.pdf]

Suppression of radiation-induced point defects by rhenium and osmium interstitials in tungsten

T. Suzudo
Center for Computational Science and e-Systems, Japan Atomic Energy Agency
2-4 Shirane Shirakata Tokai-mura, 319-1195, Japan

A. Hasegawa
Department of Quantum Science and Energy Engineering, Tohoku University
6-6-01-2 Aramaki-aza-Aoba Aoba-ku Sendai, 980-8579, Japan
(Dated: September 21, 2016)

I. DERIVATION OF BINDING ENERGY BETWEEN POINT DEFECTS AND SOLUTE ATOMS

The total energy of each case was derived after both the atomic positions and the volume of the applied supercell were fully relaxed; the convergence criterion was set at 10^{-4} eV in the total energy.

The formation energy of a vacancy was evaluated using

$$E_f^{Vac} = E_{tot}^{Vac}(N-1) - \frac{N-1}{N}E_{tot}(N), \tag{1}$$

where $E_{tot}^{Vac}(N-1)$ is the total energy of a supercell composed of $N-1$ W atoms and one vacancy; $E_{tot}(N)$ is the energy of a supercell composed of N W atoms without defects.

The formation energy of an SIA was similarly evaluated using

$$E_f^{SIA} = E_{tot}^{SIA}(N+1) - \frac{N+1}{N}E_{tot}(N), \tag{2}$$

where $E_{tot}^{SIA}(N+1)$ is a total energy of the supercell composed of $N+1$ W atoms including one SIA.

The formation energy of a substitutional solute atom was evaluated using

$$E_f^{Sub-\alpha} = E_{tot}^{Sub-\alpha}(N-1) - \frac{N-1}{N}E_{tot}(N) - E_{coh}^{\alpha}, \tag{3}$$

where $E_{tot}^{Sub-\alpha}(N-1)$ is a total energy of a supercell composed of $N-1$ W atoms and one substitutional α atom (α is Re or Os); E_{coh}^{α} is a cohesive energy of the α element.

The formation energy of an interstitial solute atom was similarly evaluated using

$$E_f^{Int-\alpha} = E_{tot}^{Int-\alpha}(N) - E_{tot}(N) - E_{coh}^{\alpha}, \tag{4}$$

where $E_{tot}^{Int-\alpha}(N)$ is a total energy of supercell composed of N W atoms and an interstitial α atom.

The energy of binding of a solute atom to a vacancy was evaluated using

$$E_b^{\alpha,Vac.} = E_f^{Sub-\alpha} + E_f^{Vac} - E_f^{Sub-\alpha,Vac}, \tag{5}$$

where $E_f^{Sub-\alpha,Vac}$ is the formation energy of a defect composed of a substitutional α and a neighboring vacancy; we considered only the first and second nearest neighbor positions. The energy of binding of a solute atom to an SIA was evaluated using

$$E_b^{\alpha,SIA} = E_f^{Sub-\alpha} + E_f^{SIA} - min(E_f^{Int-\alpha}), \tag{6}$$

where $min(E_f^{Int-\alpha})$ is the formation energy of the most stable α-solute interstitial atom. Note that any binding energies used do not depend upon the cohesive energy of the solute elements.
